# A Critical Role for Mucosal-Associated Invariant T Cells as Regulators and Therapeutic Targets in Systemic Lupus Erythematosus

**DOI:** 10.3389/fimmu.2019.02681

**Published:** 2019-11-29

**Authors:** Goh Murayama, Asako Chiba, Hitoshi Suzuki, Atsushi Nomura, Tomohiro Mizuno, Taiga Kuga, Shinji Nakamura, Hirofumi Amano, Sachiko Hirose, Ken Yamaji, Yusuke Suzuki, Naoto Tamura, Sachiko Miyake

**Affiliations:** ^1^Department of Immunology, Juntendo University School of Medicine, Tokyo, Japan; ^2^Department of Internal Medicine and Rheumatology, Juntendo University School of Medicine, Tokyo, Japan; ^3^Department of Nephrology, Juntendo University Faculty of Medicine, Tokyo, Japan; ^4^Laboratory of Morphology and Image Analysis, Research Support Center, Juntendo University School of Medicine, Tokyo, Japan; ^5^Department of Biomedical Engineering, Toin Human Science and Technology Center, Toin University of Yokohama, Yokohama, Japan

**Keywords:** MAIT cell, innate lymphocyte, MR1 ligand, lupus, T-B cell interaction

## Abstract

Mucosal-associated invariant T (MAIT) cells are a subset of innate-like lymphocytes that are restricted by major histocompatibility complex-related molecule 1 (MR1). In this study, we investigated the role of MAIT cells in the pathogenesis of lupus in FcγRIIb^−/−^*Yaa* mice, a spontaneous animal model of lupus. Using two approaches of MAIT cell deficiency, MR1 knockout animals and a newly synthesized inhibitory MR1 ligand, we demonstrate that MAIT cells augment the disease course of lupus by enhancing autoantibody production and tissue inflammation. MR1 deficiency reduced germinal center responses and T cell responses in these mice. Suppression of MAIT cell activation by the inhibitory MR1 ligand reduced autoantibody production and lupus nephritis in FcγRIIb^−/−^*Yaa* mice. MAIT cells directly enhanced autoantibody production by B cells *in vitro*. Our results indicate the contribution of MAIT cells to lupus pathology and the potential of these cells as novel therapeutic targets for autoimmune diseases such as lupus.

## Introduction

Mucosal-associated invariant T (MAIT) cells are a subset of innate-like T lymphocytes restricted by major histocompatibility complex-related molecule 1 (MR1) (1) and display innate-like properties. The majority of MR1-restricted MAIT cells express the semi-invariant T cell receptor (TCR) α chain Vα7.2-Jα33 in humans and Vα19-Jα33 in mice. MAIT cells develop in the thymus and are selected by double positive thymocytes in a MR1 dependent manner (1–3). MAIT cells recognize non-peptide antigens presented by the non-polymorphic MR1 molecules that include microbially-derived vitamin B2 (riboflavin) derivatives with MAIT cell-activating activity and vitamin B9 (folic acid) derivatives that are non-activating (4). Similar to other innate lymphocytes, including invariant natural killer T (iNKT) cells, MAIT cells respond very rapidly upon activation by TCR signaling or cytokine stimulation in the absence of exogenous antigens (5–8). Activated MAIT cells produce cytokines, including interferon (IFN)-γ, TNF-α, and IL-17 as well as cytotoxic granzyme and perforin (9–12). Therefore, MAIT cells have been speculated to play important roles in the first-line defense against microbial pathogens.

Originally, MAIT cells were named after their preferential location in mucosal tissues (2). More recent studies revealed that MAIT cells are enriched in other tissues and in the peripheral blood of humans (13, 14). Because of their characteristic abundance and cytokine-producing capacity, MAIT cells have attracted attention for their roles in various types of immune responses and diseases. MAIT cells are associated with an increasing number of diseases of microbial, autoimmune, metabolic, and cancerous origin (15–17). Previously, our group demonstrated that MR1 deficiency exacerbated the severity of experimental autoimmune encephalomyelitis, an animal model of multiple sclerosis (18). Because MR1 is essential for the thymic development of MAIT cells (1–3), they were thought to play a protective role in this model. Other studies showed that MAIT cells contribute to the suppression of the severity of inflammatory colitis and the development of type 1 diabetes (19, 20). By contrast, MAIT cells appear to contribute to tissue inflammation in arthritis models and in the pancreas of NOD mice after the onset of diabetes (5, 20).

Systemic lupus erythematosus (SLE) is a systemic autoimmune disease characterized by the production of autoantibodies to nuclear antigens. Autoantibodies form immune complexes that are deposited in tissues and cause tissue inflammation in various organs, including the skin, kidneys, joints, and central nervous system. Despite recent advances in the treatment of SLE, therapeutic options remain limited. Previously, we reported that although the frequency of MAIT cells was reduced in the peripheral blood of patients with SLE, the MAIT cells present were activated to a greater extent than those in healthy individuals, and their activated status positively correlated with disease activity (6). These findings led us to investigate the role of MAIT cells in lupus pathology.

By crossing FcγRIIb^−/−^*Yaa* mice (21), a spontaneous lupus mouse model, to MR1-deficient mice that lack MAIT cells, the current study demonstrates that MAIT cell deficiency results in reduced disease severity, as shown by decreased autoantibody production and lower glomerulonephritis scores, and that these effects are accompanied by reduced germinal center responses as well as reduced T cell and innate T cell responses in MR1-deficient lupus mice. We synthesized a new non-stimulatory MR1 ligand that inhibits MAIT cell activation, and demonstrated that the treatment of mice with the MR1 ligand reduced autoantibody production and the severity of lupus nephritis. We further showed that MAIT cells enhanced autoantibody production by B cells *in vitro* dependent on CD40L-CD40 and TCR pathways. Inhibition of MAIT cell activation by using an inhibitory MR1 ligand reduced autoantibody production by B cells. These findings highlight the crucial roles of MAIT cells in the pathogenesis of SLE and the potential of these cells as a therapeutic target of systemic autoimmune diseases, including SLE.

## Materials and Methods

### Confocal Microscopy Analysis of Human Kidney Samples

Renal biopsies were categorized into six pathological classes (I–VI) or a combination of these classes according to the ISN/RPS classification. Detection of MAIT cells in kidney biopsy samples was performed on acetone-fixed snap-frozen sections. The antibody panel included anti-CD3 (polyclonal rabbit; Abcam), anti-IL-18Rα (polyclonal goat IgG; R&D Systems), and anti-Vα7.2 (mouse IgG; BioLegend), which were detected by their respective secondary antibodies (anti-rabbit-IgG-Alexa647 and donkey anti-mouse-IgG-Alexa488; Molecular Probes, donkey anti-goat-IgG-Alexa594; Jackson ImmunoResearch, respectively). We defined MAIT cells as CD3^+^Vα7.2^+^IL-18Rα^+^DAPI^+^ cells. Analyses were performed using a TCS SP5 confocal microscope (Leica).

### Mice

Mice were maintained under specific pathogen-free conditions in accordance with the institutional guidelines of Juntendo University. FcγRIIb^−/−^*Yaa* mice were crossed to MR1^−/−^ mice to generate MR1^−/−^FcgRIIb^−/−^*Yaa* mice. MR1^−/−^ FcgRIIb^−/−^*Yaa* mice were genotyped by PCR, as previously described (2). Vα19-Jα33 TCR-transgenic (Vα19iTg) mice, originally provided by Dr. Shimamura (Teikyo Heisei University, Tokyo, Japan), were crossed with Cd1d1^−/−^ C57BL/6J mice for more than 10 generations. C57BL/6J mice were obtained from Sankyo Labo Service Corporation, Inc.

### Flow Cytometry

Splenocytes were isolated from the spleen by homogenization, and cleared of erythrocytes by ammonium-chloride-potassium lysing buffer. Renal mononuclear cells were isolated from kidneys using Multi Tissue Dissociation Kits, gentleMACS Dissociator (Miltenyi Biotec), and Percoll density-gradient centrifugation. The cells were stained using the Zombie Green Fixable Viability Kit (BioLegend) and then incubated with combinations of the following monoclonal antibodies: anti-BCl-6-PE-Cy7, anti-CD8a-V500, anti-CD4-APC-H7, anti-B220-APC-Cy7, anti-CD25-BV510 (all from BD Biosciences), anti-F4/80-FITC, anti-CD3-PE-CF594, anti-TCRγδ-PerCp-Cy5.5, anti-CD44-Alexa700, anti-CD69-PE-Cy7, anti-CD62L-BV570, anti-GL7-PerCp-Cy5.5, anti-CD44-FITC, anti-CD138-BV605, anti-B220-PE, anti-CD19-APC, anti-CD3e-BV421, anti-CD185(CXCR5)-BV421, anti-CD3-FITC, anti-CD3-Alexa700, anti-CD69-BV605, anti-ICOS-BV605 (all from BioLegend), anti-CD95-FITC, and anti-CD279(PD-1)-APC(all from eBioscience). mCD1d tetramers loaded with PBS-57-APC and mMR1 tetramers loaded with 5-OP-RU or 6-FP-BV421 were used (NIH tetramer core facility at Emory University). After staining the cell-surface antigens, intracellular staining was performed using the BD Cytofix/Cytoperm Fixation/Permeabilization Solution Kit (BD Biosciences) and anti-FOXP3-PerCP-Cy5.5 monoclonal antibody (eBioscience). Data were acquired on a FACS LSR Fortessa (BD Biosciences), and the percentages of each cell population and mean fluorescence intensity were analyzed using FlowJo software (TreeStar Inc.).

### ELISA

The serum level of anti-double-stranded (ds) DNA antibodies was measured using an ELISA kit (Shibayagi Co., Ltd.). The levels of anti-ds DNA IgG and anti-dsDNA Ig (Total A+G+M) and total IgG in culture supernatants were measured using an ELISA kit (Alpha Diagnostic International and Thermo Fisher Scientific, respectively).

### Measurement of Proteinuria

Urinary albumin levels were measured by DCA 2000 (Siemens).

### Histopathologic Analysis of Kidneys From FcγRIIb^−/−^*Yaa* Mice

Tissue sections of kidneys were fixed with 10% formalin, embedded in paraffin, and stained with periodic acid-Schiff (PAS). Pathological scores for glomerulonephritis were defined as the mean of scores from at least 50 glomeruli. Scoring was as follows: normal = 0, cell proliferation or infiltration = 1, membranoproliferation, lobulation, or hyaline deposition = 2, and crescent formation or global hyalinosis = 3.

### Confocal Immunofluorescence Microscopy Analysis of Kidneys From FcγRIIb^−/−^*Yaa* Mice

Frozen kidney sections were incubated with FITC-anti-IgG (Southern Biotech Birmingham) or FITC-anti-C3 (MP Biomedicals) and then mounted with Fluoromount/Plus (Diagnostic BioSystems). All samples were visualized using a FM1000D confocal laser scanning microscope (Olympus), and images were captured and analyzed using the FV10-ASW viewer (Olympus). The mean fluorescence intensity (MFI) of FITC was calculated using ImageJ software.

### Intracellular Staining of Cytokines and CD40L

Cells were cultured in 96-well flat-bottom plates in RPMI 1640 supplemented with 10% fetal bovine serum, 2 mM L-glutamine, 50 U/mL penicillin, and 50 μg/mL streptomycin (all from Thermo Fisher Scientific). Cells were stimulated with PMA (50 ng/mL, Sigma-Aldrich) and ionomycin (1 μg/mL, Sigma-Aldrich) for 4 h. GolgiPlug (0.67 μg/mL, BD Biosciences) was added during the final 2 h. After the cell-surface antigens were stained, intracellular cytokines were stained using the BD Cytofix/Cytoperm Fixation/Permeabilization Solution Kit (BD Biosciences), anti-TNF-PE-Cy7, anti-IFNγ-PE-Cy7, anti-IL-17-PE-Cy7, and anti-CD40L-PE-Cy7 mAb (all from BioLegend) or their isotype control antibodies.

### Treatment With MR1 Ligands

7-methyl-8-D-ribityllumazine (RL-7-Me) was synthesized as previously described (22). Isobutyryl 6-formylpterin (i6-FP) was synthesized by SundiaMediTech Company, Ltd. Splenocytes from Vα19iTgCd1d1^−/−^ mice were incubated in the presence of the activating ligand RL-7-Me and/or the suppressive ligand i6-FP for 24 h and used for flow cytometry analysis. C57BL/6J mice received 10 mg/kg or 5mg/kg of i6-FP or control vehicle orally, and 5 h later, 5 mg/kg of RL-7-Me or control vehicle was administered orally to these mice. FcγRIIb^−/−^*Yaa* and MR1^−/−^ FcγRIIb^−/−^*Yaa* mice received 10 mg/kg of i6-FP or control vehicle orally three times weekly for 4 weeks starting at 4 weeks of age.

### MAIT and B Cell Co-culture Experiments

CD19^+^ and CD14^+^ cells were deleted from splenocytes from Vα19iTg Cd1d1^−/−^ mice using anti-mouse CD19 and anti-CD14 MicroBeads (Miltenyi Biotec). MAIT cells (F4/80^−^ CD3^+^ MR1^−^tetramer^+^) were sorted from CD14^−^CD19^−^ splenocytes by MoFlo Astrios EQ (Beckman Coulter). CD19^+^ splenocytes were sorted from the spleens of FcγRIIb^−/−^*Yaa* mice using anti-mouse CD19 MicroBeads. Then, 100,000 or 20,000 MAIT cells and 20,000 CD19^+^ cells were co-cultured in 96-well V-bottom plates (Corning) with 100 ng/mL of lipopolysaccharide (LPS; InvivoGen) for 7 days. In some experiments, 10 μg/mL of anti-mouse CD154 antibody, anti-mouse CD275 antibody, anti-mouse MR1 antibody (all from BioLegend) or 10 μM i6-FP was added.

### Statistical Analysis

All data were analyzed using GraphPad Prism (GraphPad, Inc.), and differences between groups were analyzed using the Mann-Whitney U-test, the generalized Wilcoxon test, and the one-way ANOVA followed by Tukey's multiple comparison test. The significance level was set at *p* < 0.05.

### Study Approval

All participants signed informed consent, and the Research Ethics Review Committees of the Juntendo University Hospital approved the study protocol. All animal studies and procedures were approved by the Animal Experimental Committee of the Juntendo University Graduate School of Medicine.

## Results

### Activated MAIT Cells Infiltrate the Kidneys of Patients With Lupus Nephritis and FcγRIIb^−/−^*Yaa* Mice

**Figure 1:**
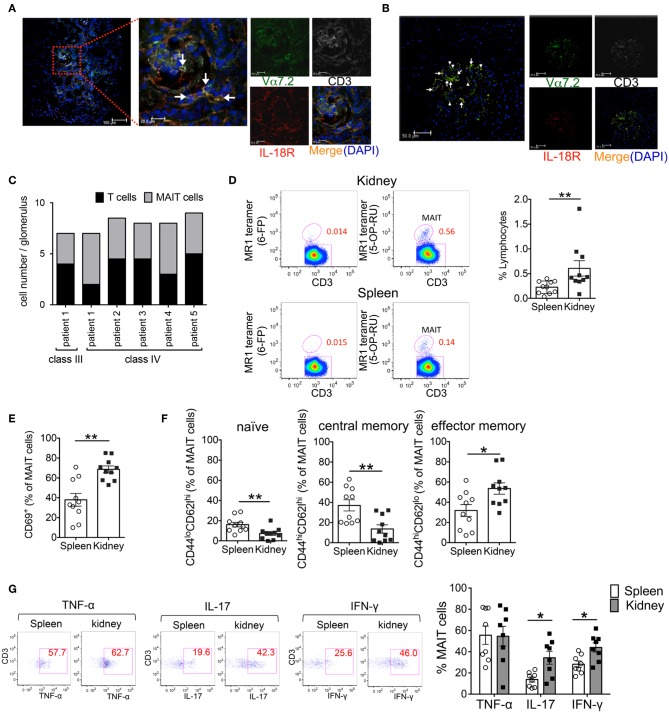
Activated MAIT cells infiltrate the kidneys of patients with lupus nephritis and FcγRIIb^−/−^*Yaa* mice. **(A–C)** To identify MAIT cells using confocal microscopy, snap-frozen kidney tissues from patients with systemic lupus erythematosus (SLE) were triple-stained with antibodies against interleukin (IL)-18Rα (red), Vα7.2 TCR (green) and CD3 (white) and cell nuclei were stained with 4′, 6-diamidino-2-phenylindole (DAPI). The white arrow denotes MAIT cells and the arrowhead denotes other CD3^+^ cells. Representative images in the tubulointerstitium (*n* = 6) **(A)** and glomerulus (*n* = 6) **(B)** are shown. Scale bars = 20 μm and 100 μm in high- and low-magnification fields, respectively, in **(A)** and 50 μm in **(B)**. Values in **(C)** are the mean cell numbers of MAIT and CD3^+^ cells per glomerulus. **(D–G)** Frequency and phenotype of MAIT cells in the spleen (*n* = 10) and kidneys (*n* = 10) from FcγRIIb^−/−^*Yaa* mice at 2 months of age. **(D)** Representative flow cytometry profiles of F4/80^−^CD3^+^TCRγδ^−^ CD1d/PBS-57 tetramer^−^ lymphocytes in the spleen and kidneys. MAIT cells were identified as F4/80^−^CD3^+^TCRγδ^−^ CD1d/PBS-57 tetramer^−^ MR1/5-OP-RU^+^ lymphocytes. Flow cytometric evaluation of the frequencies of MAIT cells **(D)** and CD69^+^, naïve, central memory and effector memory cells among MAIT cells **(E,F)**. **(G)** Flow cytometric evaluation of the frequencies of the indicated cytokine-producing cells among MAIT cells upon stimulation with PMA and ionomycin. Representative flow cytometry profiles of cytokine staining in MAIT cells are shown. Values in **(D–G)** are shown as the mean ± SEM. Each symbol represents data from an individual mouse. *p*-values in **(D–G)** were determined by two-tailed Mann–Whitney *U*-test (**p < 0.01, *p < 0.05).

Previously, we demonstrated that in SLE, the reduced frequency of MAIT cells in the peripheral blood was associated with the enhanced activation-induced cell death of these cells. Because MAIT cells are often found at the site of inflammation in diseases such as inflammatory arthritis and colitis (23–26). MAIT cell loss in the peripheral blood of SLE patients might be related to the migration of these cells into tissues. Thus, we examined whether MAIT cells infiltrated into the inflamed tissues of SLE patients. Kidney biopsy specimens from patients with lupus nephritis were evaluated by confocal microscopy for MAIT cells. The infiltration of CD3^+^ cells into the glomeruli and tubulointerstitium was observed in patients with class III and IV but not class II and V disease (Figures 1A–C, Supplementary Table 1). MAIT cells (CD3^+^Vα7.2TCR^+^IL-18Rα^+^) were present in the glomeruli and tubulointerstitium in patients with class III and IV disease (Figures 1A–C, Supplementary Table 1). In particular, MAIT cells constituted a high proportion of glomerular CD3^+^ cells (Figure 1C, Supplementary Table 1). These data suggested that MAIT cells may contribute to the pathogenesis of SLE.

We previously generated FcγRIIb^−/−^*Yaa* mice that develop glomerulonephritis accompanied by the production of various types of autoantibodies, including anti-double strand(ds) DNA antibody (21). To explore the involvement of MAIT cells in lupus nephritis, we investigated the frequency of MAIT cells and their contribution to tissue inflammation in FcγRIIb^−/−^*Yaa* mice. The frequency of MAIT cells among lymphocytes was approximately 0.23% in the spleen and 0.42% in the kidneys (Figure 1D, Supplementary Figure 1). The frequency of CD69-expressing cells among MAIT cells was significantly higher in MAIT cells in the kidneys than those in the spleen (Figure 1E). There was more effector memory (CD44^hi^CD62L^lo^) cells than naïve (CD44^lo^CD62L^hi^) or central memory (CD44^hi^CD62L^hi^) cells among MAIT cells in the kidneys compared to those in the spleen (Figure 1F). Upon stimulation, MAIT cells in the spleen and kidneys expressed high levels of cytokines including TNF-α, IL-17, and IFN-γ. The proportions of IL-17- or IFN-γ-expressing cells were higher among MAIT cells in the kidneys compared to those in the spleen (Figure 1G). We also assessed differences in the subset and cytokine producing capacity of MAIT cells between FcγRIIb^−/−^*Yaa* mice and C57BL/6J mice (Supplementary Figure 2). The frequency of splenic MAIT cells was higher in FcγRIIb^−/−^*Yaa* mice compared with C57BL/6J mice. The frequencies of central memory and effector memory MAIT cells were higher in FcγRIIb^−/−^*Yaa* mice than those in C57BL/6J mice, and conversely there were less naïve MAIT cells in FcγRIIb^−/−^*Yaa* mice. In addition, the proportions of TNF-α and IFN-γ-expressing MAIT cell were higher in FcγRIIb^−/−^*Yaa* mice than in C57BL/6J mice. These findings suggested that MAIT cells in FcγRIIb^−/−^*Yaa* mice may act as effector cells contributing to the tissue inflammation of nephritis.

### MR1 Deficiency Ameliorates the Course of Lupus in FcγRIIb^−/−^*Yaa* Mice

**Figure 2:**
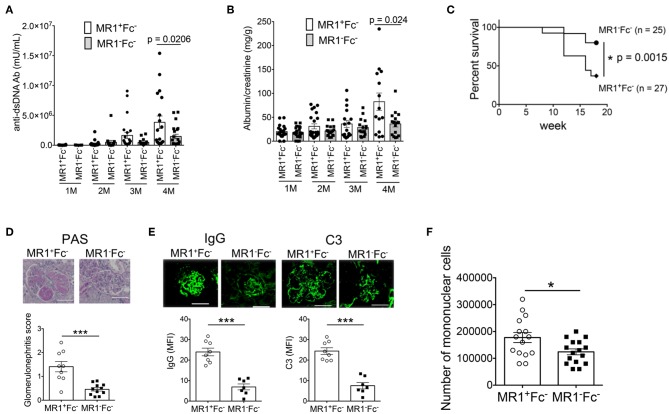
MR1 deficiency ameliorates the disease course of lupus in FcγRIIb^−/−^*Yaa* mice. Serum levels of anti-dsDNA antibody (Ab) **(A)** and levels of proteinuria **(B)** at 1, 2, 3, and 4 months of age, and survival rate **(C)** of MR1^+/+^ FcγRIIb^−/−^*Yaa* (MR1^+^Fc^−^) (*n* = 27) and MR1^−/−^ FcγRIIb^−/−^*Yaa* (MR1^−^Fc^−^) mice (*n* = 25). **(D,E)** Histopathological analysis of glomeruli in MR1^+^Fc^−^ (*n* = 9) and MR1^−^Fc^−^ (*n* = 10) mice at 2 months of age. **(D)** Representative images of kidney sections and histopathological scores. Scale bars, 50 μm. **(E)** Representative images of frozen kidney sections stained with anti-mouse IgG and C3 in glomeruli (scale bar, 50 μm). **(F)** Enumeration of mononuclear cells in the kidneys of MR1^+^Fc^−^ (*n* = 15) and MR1^−^Fc^−^ mice (*n* = 16) at 2 months of age. All data in **(A–F)** are shown as the mean ± SEM. Each symbol represents data from an individual mouse. *p*-values in A-D were determined by two-tailed Mann-Whitney U-test (***p < 0.001, *p < 0.05). The survival rate in **(C)** was assessed by a two-tailed, generalized Wilcoxon test. The *p*-value for the overall comparison is shown.

To investigate whether MAIT cells contribute to the pathological process of lupus, we crossed MAIT cell-deficient MR1 knockout mice with FcγRIIb^−/−^*Yaa* mice and compared the disease course of MR1^−/−^FcγRIIb^−/−^*Yaa* mice and littermate MR1^+/+^FcγRIIb^−/−^*Yaa* mice (henceforth referred to as MR1^−^Fc^−^ and MR1^+^Fc^−^ mice, respectively). MR1 deficiency in FcγRIIb^−/−^*Yaa* mice was assessed by PCR-based genotyping using tail DNA (Supplementary Figure 3A). We confirmed that MR1 deficiency resulted in a lack of MAIT cells by staining splenocytes from MR1^−^Fc^−^ and MR1^+^Fc^−^ mice with MR1 tetramer (Supplementary Figure 3B). Serum levels of anti-dsDNA antibody were lower in MR1^−^Fc^−^ mice than MR1^+^Fc^−^ mice at 4 months of age (Figure 2A). Both MR1^+^Fc^−^ and MR1^−^Fc^−^ mice developed proteinuria starting at 1 month of age (Figure 2B). The level of proteinuria was reduced and the survival rate was increased by MR1 deficiency in FcγRIIb^−/−^*Yaa* mice (Figures 2B,C). Histopathological analysis of kidneys revealed decreased severity of glomerulonephritis by MR1 deficiency, as shown by the decreased glomerulonephritis scores in MR1^−^Fc^−^ mice compared with MR1^+^Fc^−^ mice (Figure 2D). The deposition of IgG and C3 in the kidneys of FcγRIIb^−/−^*Yaa* mice and the infiltration of mononuclear cells into the kidneys were reduced by MR1 deficiency (Figures 2E,F). These results indicate that autoantibody production and the clinical course of lupus were ameliorated by MR1 deficiency.

### MR1 Deficiency Reduces Germinal Center Reaction in FcγRIIb^−/−^*Yaa* Mice

**Figure 3:**
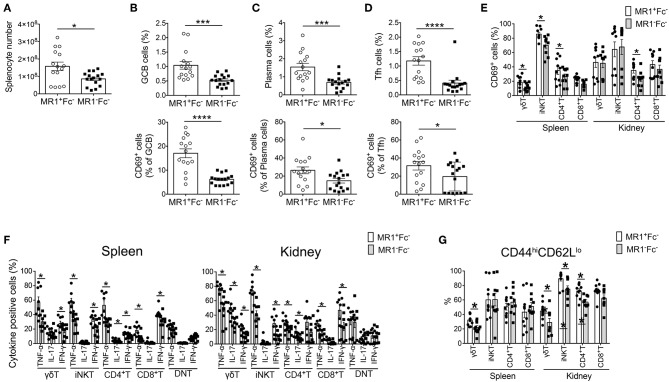
MR1 deficiency reduces the germinal center reaction and innate T and T cell responses in FcγRIIb^−/−^*Yaa* mice. **(A–D)** Cell numbers of splenocytes and flow cytometric evaluation of frequencies of the indicated subsets of B and T cells in MR1^+/+^ FcγRIIb^−/−^*Yaa* (MR1^+^Fc^−^) (*n* = 15) and MR1^−/−^ FcγRIIb^−/−^*Yaa* (MR1^−^Fc^−^) mice (*n* = 16) at 2 months of age. **(A)** Cell numbers of splenocytes. **(B)** Percentages of germinal center B cells (GCB) (B220^+^CD95^+^GL7^+^) and CD69^+^ GCB cells. **(C)** Frequencies of plasma cells (CD3^−^CD19^−^CD138^+^) and CD69^+^ plasma cells. **(D)** Frequencies of T follicular helper cells (Tfh) (CD3^+^CD4^+^Foxp3^−^CXCR5^+^PD-1^hi^) and CD69^+^ cells among Tfh cells. **(E–G)** Cell surface phenotype and function of innate T and T cells in the spleen and kidneys from MR1^+^Fc^−^ (*n* = 10) and MR1^−^Fc^−^ mice (*n* = 10) at 2 months of age. **(E)** Frequencies of CD69^+^ cells among γδT cells, iNKT cells, CD4^+^T cells, and CD8^+^T cells. **(F)** Frequencies of the indicated cytokine-producing cells among γδT, iNKT, CD4^+^T, and CD8^+^T and CD4^−^CD8^−^ double-negative (DN)T cells upon stimulation with PMA and ionomycin. **(G)** Flow cytometric evaluation of the frequencies of effector memory T cells among γδT cells, iNKT cells, CD4^+^T cells, and CD8^+^T cells. All data in **(A–G)** are shown as the mean ± SEM. Each symbol represents the value of one individual. *p*-values were determined by two-tailed Mann–Whitney *U*-test (****p < 0.0001, ***p < 0.001, *p < 0.05).

Because autoantibody production was reduced in MR1^−^Fc^−^ mice, we next assessed the effect of MR1 deficiency on B cell responses in FcγRIIb^−/−^*Yaa* mice. The number of total splenocytes in FcγRIIb^−/−^*Yaa* mice was markedly reduced by MR1 deficiency (Figure 3A). There were fewer germinal center (GC) B cells in MR1^−^Fc^−^ mice than in MR1^+^Fc^−^ mice (Figure 3B). In addition, the proportion of CD69^+^ cells among GC B cells was significantly reduced by MR1 deficiency (Figure 3B). The frequencies of plasma cells and CD69^+^ plasma cells were also decreased by MR1 deficiency in FcγRIIb^−/−^*Yaa* mice (Figure 3C). The CD44 pathway promotes the survival of plasma cells (27, 28), we found that CD44 expression was reduced in these cells from MR1^−^Fc^−^ mice compared with MR1^+^Fc^−^ mice (Supplementary Figure 3C). Although the frequencies of T follicular helper (Tfh) cells and CD69^+^ Tfh cells were reduced, those of T regulatory and T follicular helper regulatory (Tfr) cells were increased in MR1^−^Fc^−^ compared with MR1^+^Fc^−^ mice (Figure 3D, Supplementary Figure 3D). Thus, MAIT cells may promote GC reactions, resulting in augmented autoantibody production in FcγRIIb^−/−^*Yaa* lupus mice.

### MR1 Deficiency Reduces Innate T and T Cell Responses in the Spleen and Kidneys of FcγRIIb^−/−^*Yaa* Mice

Next, we analyzed the activation status and cytokine-producing capacity of these cells in the spleen and kidneys. The frequencies of CD69^+^ activated cells among γδT, iNKT, and CD4^+^T cells in the spleen and among CD4^+^T cells in the kidney were reduced by MR1 deficiency in FcγRIIb^−/−^*Yaa* mice (Figure 3E). The capacity to produce the cytokines TNF-α, IL-17, and IFN-γ was decreased in most innate T and T cells related to MR1 deficiency in FcγRIIb^−/−^*Yaa* mice (Figure 3F, Supplementary Figure 4). CD4^−^CD8^−^ double-negative (DN) T cells were implicated in the pathogenesis of lupus in humans and mice (29–33). However, MR1 deficiency did not reduce the cytokine producing capacity of DNT cells in FcγRIIb^−/−^*Yaa* mice (Figure 3F). Furthermore, the frequency of effector memory cells among γδT cells in the spleen and kidney and those among iNKT and CD4^+^T cells in the kidney were decreased in MR1^−^Fc^−^ compared with MR1^+^Fc^−^ mice (Figure 3G). However, naïve and central memory cells among γδT and NKT cells in the kidneys and naïve CD4^+^T cells in the spleen were increased in MR1^−^Fc^−^ mice (Supplementary Figure 3E). These results indicated that a deficiency of MAIT cells reduced cytokine production by innate T and T cells, and also reduced the accumulation of effector T cells in the kidneys of the FcγRIIb^−/−^*Yaa* lupus mouse model.

### Inhibition of MAIT Cell Activation Suppresses Lupus in FcγRIIb^−/−^*Yaa* Mice

**Figure 4:**
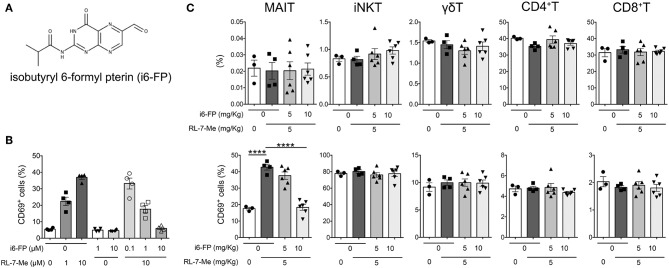
Isobutyryl 6-formyl pterin inhibits MAIT cell activation. **(A)** The chemical structure of isobutyryl 6-formyl pterin (i6-FP). **(B)** Splenocytes from Vα19iTgCd1d1^−/−^ mice were incubated in the presence of graded doses of i6-FP and/or 7-methyl-8-D-ribityllumazine (RL-7-Me) for 16 h, and the frequency of CD69^+^ MAIT cells was analyzed by flow cytometry. Frequencies of CD69^+^ cells among MAIT cells are shown. The results are representative of two separate experiments. **(C)** i6-FP (5 or 10 mg/kg) or control vehicle was administered orally to C57BL/6J mice 5 h prior to the oral administration of RL-7-Me (5 mg/kg) or control vehicle. After 16 h, frequencies and CD69 expression of each innate T and T cell subset was analyzed by flow cytometry. Frequencies of γδT, iNKT, MAIT, CD4^+^T and CD8^+^T cells, and CD69^+^ cells among each innate T and T cell subset are shown. All data in **(B,C)** are shown as the mean ± SEM. Each symbol represents data from individual animals. *p*-values in **(C)** were determined by two-tailed, one-way ANOVA followed by Tukey's multiple comparison tests (****p < 0.0001).

Because our findings suggested a crucial role for MAIT cells in lupus pathology, we investigated whether the inhibition of MAIT cell activation could effectively suppress lupus. To this end, we hypothesized that 6-formylpterin (6-FP), a vitamin B9 metabolite that binds to the MR1 molecule but does not cause MAIT cell activation (4, 22), would inhibit MAIT cell activation. Because the inhibitory potency of 6-FP is weak (34, 35), we synthesized an analog of 6-FP, isobutyryl 6-FP (i6-FP) (Figure 4A), and tested its inhibitory effects on MAIT cell activation. Splenocytes from Vα19iTg Cd1d^−/−^ mice that contain a high frequency of MAIT cells were treated with a stimulatory MR1 ligand, 7-methyl-8-D-ribityllumazine (RL-7-Me), in the presence or absence of i6-FP. Treatment with RL-7-Me resulted in the activation of MAIT cells as observed by the increased frequency of CD69^+^ MAIT cells; i6-FP inhibited RL-7-Me-induced MAIT cell activation in a dose-dependent manner (Figure 4B). To check whether the inhibitory effect of i6-FP on MAIT cell activation also occurred *in vivo*, mice were pretreated with i6-FP (5 or 10 mg/kg) orally followed by the oral administration of RL-7-Me (5 mg/kg), and CD69 expression on splenic MAIT cells and other T cell subsets was evaluated. The frequency of MAIT cells among lymphocytes did not change with the administration of MR1 ligands (Figure 4C). The frequency of CD69^+^ MAIT cells was increased in mice treated with RL-7-Me, and pretreatment with 10 mg/kg of i6-FP inhibited the RL-7-Me-induced increase of CD69^+^ MAIT cells (Figure 4C). Administration of MR1 ligands had no effect on other T cell subsets (Figure 4C). These results indicate that i6-FP suppresses stimulatory MR1 ligand-induced MAIT cell activation *in vivo*.

**Figure 5:**
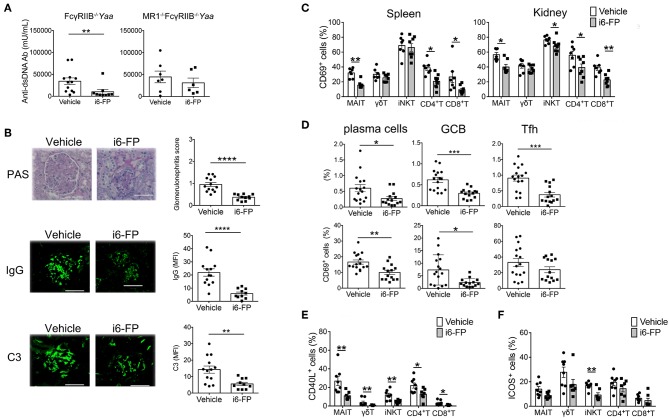
i6-FP administration suppresses lupus in FcγRIIb^−/−^*Yaa* mice. FcγRIIb^−/−^*Yaa* (A-E) and MR1^−/−^ FcγRIIb^−/−^*Yaa* (MR1^−^Fc^−^) **(A)** mice were treated orally with i6-FP (10 mg/kg) (*n* = 14, *n* = 6, respectively) or control vehicle (*n* = 16, *n* = 7, respectively) three times weekly starting at 4 weeks of age. **(A)** Serum levels of anti-dsDNA antibody (Ab) in FcγRIIb^−/−^*Yaa* and MR1^−^Fc^−^ mice were determined by ELISA. **(B)** Histopathological findings of glomeruli. Representative images of kidney sections and histopathological scores are shown. Scale bars, 50 μm. Representative images of frozen kidney sections stained with anti-mouse IgG and C3 in glomeruli (scale bar, 50 μm). **(C)** CD69 expression of innate T and T cells in the spleen and kidneys from FcγRIIb^−/−^*Yaa* mice at 2 months were analyzed by flow cytometry. Percentage of CD69 among γδT cells, iNKT cells, MAIT cells, CD4^+^T cells and CD8^+^T cells are shown. **(D)** Flow cytometric evaluation of the frequencies of germinal center B cells (GCB), plasma cells and T follicular helper cells (Tfh) and percentages of CD69 among these B and T cell subsets. **(E)** Flow cytometric evaluation of the frequencies of CD40L-positive cells among the indicated innate T and T cell subsets upon stimulation with PMA and ionomycin. **(F)** Frequencies of ICOS-positive cells among the indicated innate T and T cell subsets. All data in **(A–F)** are shown as the mean ± s.e.m. Each symbol represents data from individual mice. *p*-values in a-d were determined by two-tailed Mann–Whitney *U*-test (****p < 0.0001, ***p < 0.001, **p < 0.01, *p < 0.05).

Next, we investigated whether the inhibition of MAIT cell activation by i6-FP (10 mg/kg) suppressed lupus in FcγRIIb^−/−^*Yaa* mice. The serum levels of anti-dsDNA antibody and the severity of lupus nephritis were reduced by treatment with i6-FP, as shown by the reduced glomerulonephritis score in these mice (Figure 5A-left panel, Figure 5B-top panel). However, i6-FP administration to MR1^−^Fc^−^ mice failed to reduce the serum levels of anti-dsDNA (Figure 5A-right panel). IgG and C3 deposition in glomeruli were also suppressed in i6-FP-treated FcγRIIb^−/−^*Yaa* mice (Figure 5B-middle and bottom panels).

i6-FP treatment reduced the proportions of CD69^+^ cells among most innate T and T cells in the spleen and kidneys (Figure 5C). i6-FP-induced suppression of autoantibody production was associated with reduced frequencies of plasma cells, germinal center B cells, and Tfh cells in the spleen (Figure 5D). The percentages of CD69^+^ cells were also decreased among plasma cells and germinal center B cells, suggesting the suppression of germinal center responses by i6-FP treatment in these mice (Figure 5D). i6-FP treatment in MR1^−^Fc^−^ mice did not suppress the activation of innate T cells and T cells in the spleen and kidneys, and did not reduce the frequencies of plasma cells, germinal center B cells, or Tfh cells in the spleen (Supplementary Figure 5). CD40L expression after stimulation with PMA/ionomycin was decreased in all innate T and T cells from i6-FP-treated FcγRIIb^−/−^*Yaa* mice (Figure 5E). The frequency of ICOS^+^ cells among iNKT cells, but not other innate T and T cells, was reduced in i6-FP-treated FcγRIIb^−/−^*Yaa* mice (Figure 5F). The capacity to produce TNF-α, IL-17, and IFN-γ was decreased in most innate T and T cells in FcγRIIb^−/−^*Yaa* mice treated with i6-FP (Supplementary Figure 6).

Taken together, the inhibition of MAIT cell activation by i6-FP treatment suppressed the activation of innate T and T cell subsets, which resulted in the reduction of germinal center responses and autoantibody production in FcγRIIb^−/−^*Yaa* mice.

### MAIT Cells Enhance Autoantibody Production by B Cells Dependent on the CD40-CD40L and TCR Pathways

**Figure 6:**
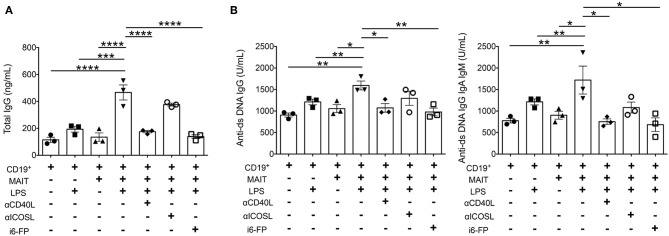
MAIT cells enhance autoantibody production by B cells dependent on CD40-CD40L and TCR pathways. B cells from FcγRIIb^−/−^*Yaa* mice were stimulated with lipopolysaccharide (LPS) in the presence or absence of MAIT cells at a ratio of 1:5, blocking antibodies against CD40L, ICOS or i6-FP (10 μM). ELISA results of total IgG **(A)**, and anti-dsDNA IgG and anti-ds DNA IgG+A+M **(B)** in culture supernatants. Each symbol represents data from each experiment. *p*-values were determined by two-tailed, one-way ANOVA followed by Tukey's multiple comparison tests (****p < 0.0001, ***p < 0.001, **p < 0.01, *p < 0.05).

Because autoreactive B cell responses were reduced by MR1 deficiency or i6-FP treatment in FcγRIIb^−/−^*Yaa* mice, we next investigated whether MAIT cells directly activate B cells to produce autoantibodies. B cells from FcγRIIb^−/−^*Yaa* mice were stimulated with lipopolysaccharide (LPS) in the presence or absence of MAIT cells, and the levels of antibodies in the culture supernatants were measured. The addition of MAIT cells to B cells at ratios of 1:1 or 5:1 enhanced IgG production by B cells stimulated with LPS (Figure 6A, Supplementary Figure 7). Blockade of CD40L-CD40 interactions or the MR1-TCR pathway partially reduced total IgG production by activated B cells (Figure 6A, Supplementary Figure 7). We also assessed the effect of MAIT cells on autoantibody production by B cells. The production of anti-dsDNA IgG and anti-dsDNA IgG+A+M by activated B cells was also enhanced by MAIT cells, and this was markedly reduced by blocking CD40L-CD40 or the MR1-TCR pathway (Figure 6B, Supplementary Figure 7). These results indicate that MAIT cells contribute to autoreactive B cell responses through direct interaction with B cells.

## Discussion

Because of the unique features of MAIT cells including their abundance, migratory capability, and ability to produce proinflammatory cytokines and cytotoxic proteins, their role in various types of immune diseases, including lupus, has generated increasing interest (16, 17). However, role of MAIT cells in lupus pathology is poorly understood.

Previously, we reported that the frequency of MAIT cells in the peripheral blood of SLE patients was markedly decreased compared with that of other innate-like T cells, and that the activated status of MAIT cells positively correlated with disease activity in SLE (6). MAIT cell activation was associated with the enhanced MR1 antigen-presenting capacity of monocytes and elevated cytokines, such as IL-18 and IFN-α, which activate MAIT cells in a TCR-independent manner and are overproduced in patients with SLE (6, 36). Thus, we speculated that altered immune responses related to dysregulated MAIT cell responses may contribute to pre-existing susceptibility to autoimmunity. MR1 deficiency influenced autoantibody production and the disease severity of lupus in FcγRIIb^−/−^*Yaa* mice, suggesting that MAIT cells are responsible for enhanced autoimmune responses in lupus. Interestingly, the frequency of MAIT cells in human peripheral blood is highest among women of fertile age and is significantly lower in elderly individuals (37). Given that the peak incidence of SLE occurs during the reproductive years, MAIT cells might be involved in the acceleration of autoimmune responses in humans.

MR1 deficiency decreased autoantibody production, which was associated with a reduced germinal center reaction. Human MAIT cells have been shown to expand plasmablasts *in vitro* (38). Other innate-like T cells, including γδT cells and iNKT cells, are known to provide help to B cells (39–42). Recently, γδT cells were shown to promote Tfh cell differentiation through antigen presentation and production of Wnt ligands, and reduced autoantibody production and glomerulonephritis were observed in γδT cell-deficient mice in a pristine model of lupus (41). Depletion of human iNKT cells reduced the secretion of anti-dsDNA antibody by lupus peripheral blood mononuclear cells (43). We demonstrated that MAIT cells enhanced autoantibody production by B cells *in vitro* dependent on CD40L-CD40 interactions and TCR pathways. Because both MR1 deficiency and i6-FP administration reduced Tfh cell responses and innate T and T cell responses, MAIT cells may influence autoreactive B cell responses through these innate T and T cells as well as direct interactions with B cells.

The addition of MAIT cells to B cells at ratios of 1:1 or 5:1 enhanced antibody production by B cells. Considering the low frequency of MAIT cells in mice, further investigations are needed to reveal the mechanism by which MAIT-B cell interactions occur. It would be interesting to study whether MAIT cells migrate to B-cell follicles and how MAIT cells encounter autoreactive B cells.

MAIT cells produce cytokines, including IFN-γ, TNF-α, and IL-17, and the levels of these cytokines were reported to be elevated in patients with SLE. Increased serum levels of IFN-γ and IFN-α precede autoantibody production and classification of SLE (44). These cytokines also cause tissue inflammation. MAIT cells from FcγRIIb^−/−^*Yaa* mice produced high amounts of these cytokines upon activation and a deficiency of MAIT cells resulted in reduced production of these cytokines by other innate T and T cells. Therefore, MAIT cells may enhance autoantibody production and tissue inflammation by producing inflammatory cytokines and influencing other innate T and T cells.

As previously reported by us and others, the frequency of MAIT cells is reduced in the peripheral blood of SLE patients (6) and peripheral blood MAIT cells are decreased in patients with inflammatory arthritis, including ankylosing spondylitis (AS) and rheumatoid arthritis (RA), and inflammatory bowel diseases (IBD) (23–26, 45). MAIT cells have the capacity to migrate into sites of inflammation, and we showed that MAIT cells constitute a large proportion of CD3^+^ cells in the kidneys of patients with class III and IV lupus nephritis. In different types of lupus nephritis, intracapillary immune complex deposition and active or chronic lesions with endo- or extracapillary proliferation are the hallmarks of lupus nephritis class III and IV. MAIT cells were mainly identified in the glomeruli of class III and IV lupus nephritis. Thus, infiltration of MAIT cells may be involved in severe kidney injury. MAIT cells were also shown to be enriched in the synovial fluid of patients with AS and RA, as well as in the gut tissues of patients with IBD. These findings raise the question of whether MAIT cells might be a therapeutic target in such autoimmune diseases. MAIT cells are usually present in low numbers in most laboratory strains of mice, and as predicted, the percentages of MAIT cells in the spleen and kidneys of FcγRIIb^−/−^*Yaa* mice were low and constituted <1% of lymphocytes. Despite this low frequency, MAIT cell deficiency and suppression of their activation by MR1 ligands had a substantial impact on other immune cells and tissue inflammation in lupus. Therefore, inhibition of MAIT cell activation may suppress such tissue inflammation even in patients with low MAIT cell frequency in the peripheral blood.

Vitamin B2 (riboflavin) precursor derivatives presented by MR1 molecules activate MAIT cells. Enzymes required for riboflavin biosynthesis are deficient in some bacterial species, including *Lactobacillus*, which belongs to *Firmicutes*. Notably, SLE patients have a lower *Firmicutes/Bacteroides* ratio than healthy individuals (46). The gut microbiota in lupus-prone MRL/lpr mice also showed a decrease in the family *Lactobacillaceae*, which belongs to *Firmicutes* (47). Therefore, dysbiosis may be associated with the activation of MAIT cells in SLE patients and lupus-prone mice. It would be interesting to investigate whether fecal microbiota transplantation from SLE patients in mouse models of lupus results in MAIT cell activation and disease exacerbation.

The levels of anti-dsDNA antibody were comparable between MR1^−^Fc^−^ and MR1^+^Fc^−^ mouse groups at 1, 2, and 3 months of age. However, i6-FP treatment in FcγRIIb^−/−^*Yaa* mice starting at 4 weeks of age reduced the anti-dsDNA antibody levels and the severity of glomerulonephritis at 8 weeks of age. Reasons for the profound effect of i6-FP treatment on the disease course is currently unknown. We speculate that the difference in the effects between MR1 deficiency and i6-FP treatment on the disease course of lupus may be due to the double-sided functions of MAIT cells on autoimmune responses. We previously reported that MR1 deficiency exacerbated EAE, but inflammatory responses were reduced in arthritis models using MR1-deficient mice (5, 18). Rouxel et al. demonstrated that, although MAIT cells play an important role in maintaining gut integrity and the suppression of anti-islet responses in NOD mice, activated MAIT cells had cytotoxic effects on islet cells after the onset of diabetes (20). These opposite effects on autoimmune responses might occur during the early phase of lupus in FcγRIIb^−/−^*Yaa* mice. MAIT cells might contribute to the suppression of autoimmune responses in the subclinical stage of lupus. Thus, MR1 deficiency had little effect on anti-dsDNA antibody levels for the first 3 months of age, and the inhibition of MAIT cell activation with i6-FP treatment had beneficial effects on the disease course of lupus.

This study uncovered the roles of MAIT cells in lupus pathology and the potential of these cells as therapeutic targets for the treatment of SLE. Using two approaches to obtain MAIT cell deficiency, MR1 knockout animals and use of an inhibitory MR1 ligand, we demonstrate that MAIT cells augment the disease course of lupus by enhancing autoantibody production and tissue inflammation. The accumulation of MAIT cells in inflamed kidney tissues in mice and lupus patients suggested that MAIT cells function as effector cells in nephritis. Thus, blocking MAIT cell activation may be beneficial for the inhibition of autoantibody production and to suppress tissue inflammation even after autoantibodies have been produced. Together with our previous study that reported a correlation of MAIT cell activation with disease activity in SLE (6), these findings suggest that MAIT cells may be attractive targets for the development of new strategies to treat autoimmune diseases.

## Data Availability Statement

All data needed to evaluate the conclusions in the paper are present in the article/Supplementary Material. Additional data and methods related to this paper may be requested from the authors.

## Ethics Statement

All participants signed informed consent, and the Research Ethics Review Committees of the Juntendo University Hospital approved the study protocol. All animal studies and procedures were approved by the Animal Experimental Committee of the Juntendo University Graduate School of Medicine.

## Author Contributions

GM, AC, and SM designed the research and wrote the manuscript. GM, AN, TM, TK, and SN performed experiments and data analysis. HS, HA, KY, YS, and NT characterized patients and provided human samples. HA and SH provided FcγRIIb^−/−^*Yaa* mice. All authors contributed to the interpretation of the data and preparation of the manuscript.

### Conflict of Interest

Some results in this study were used for a patent application submitted by Juntendo University. The authors declare that the research was conducted in the absence of any commercial or financial relationships that could be construed as a potential conflict of interest.

## References

[1] Tilloy F Treiner E Park SH Garcia C Lemonnier F de la Salle H. An invariant T cell receptor alpha chain defines a novel TAP-independent major histocompatibility complex class Ib-restricted alpha/beta T cell subpopulation in mammals. J Exp Med. (1999) 189:1907–21. 10.1084/jem.189.12.1907 10377186 PMC2192962

[2] Treiner E Duban L Bahram S Radosavljevic M Wanner V Tilloy F Selection of evolutionarily conserved mucosal-associated invariant T cells by MR1. Nature. (2003) 422:164–9. 10.1038/nature01433 12634786

[3] Seach N Guerri L Le Bourhis L Mburu Y Cui Y Bessoles S. Double-positive thymocytes select mucosal-associated invariant T cells. J Immunol. (2013) 191:6002–9. 10.4049/jimmunol.1301212 24244014

[4] Kjer-Nielsen L Patel O Corbett AJ Le Nours J Meehan B Liu L. MR1 presents microbial vitamin B metabolites to MAIT cells. Nature. (2012) 491:717–23. 10.1038/nature11605 23051753

[5] Chiba A Tajima R Tomi C Miyazaki Y Yamamura T Miyake S. Mucosal-associated invariant T cells promote inflammation and exacerbate disease in murine models of arthritis. Arthritis Rheum. (2012) 64:153–61. 10.1002/art.33314 21904999

[6] Chiba A Tamura N Yoshikiyo K Murayama G Kitagaichi M Yamaji K. Activation status of mucosal-associated invariant T cells reflects disease activity and pathology of systemic lupus erythematosus. Arthritis Res Ther. (2017) 19:58. 10.1186/s13075-017-1257-5 28288675 PMC5348792

[7] van Wilgenburg B Scherwitzl I Hutchinson EC Leng T Kurioka A Kulicke C. MAIT cells are activated during human viral infections. Nat Commun. (2016) 7:11653. 10.1038/ncomms11653 27337592 PMC4931007

[8] Ussher JE Bilton M Attwod E Shadwell J Richardson R de Lara C. CD161++ CD8+ T cells, including the MAIT cell subset, are specifically activated by IL-12+IL-18 in a TCR-independent manner. Eur J Immunol. (2014) 44:195–203. 10.1002/eji.201343509 24019201 PMC3947164

[9] Rahimpour A Koay HF Enders A Clanchy R Eckle SB Meehan B. Identification of phenotypically and functionally heterogeneous mouse mucosal-associated invariant T cells using MR1 tetramers. J Exp Med. (2015) 212:1095–108. 10.1084/jem.20142110 26101265 PMC4493408

[10] Dusseaux M Martin E Serriari N Peguillet I Premel V Louis D. Human MAIT cells are xenobiotic-resistant, tissue-targeted, CD161hi IL-17-secreting T cells. Blood. (2011) 117:1250–9. 10.1182/blood-2010-08-303339 21084709

[11] Miyazaki Y Miyake S Chiba A Lantz O Yamamura T. Mucosal-associated invariant T cells regulate Th1 response in multiple sclerosis. Int Immunol. (2011) 23:529–35. 10.1093/intimm/dxr047 21712423

[12] Le Bourhis L Martin E Péguillet I Guihot A Froux N Coré M. Antimicrobial activity of mucosal-associated invariant T cells. Nat Immunol. (2010) 11:701–8. 10.1038/ni.1890 20581831

[13] Martin E Treiner E Duban L Guerri L Laude H Toly C. Stepwise development of MAIT cells in mouse and human. PLoS Biol. (2009) 7:e54. 10.1371/journal.pbio.1000054 19278296 PMC2653554

[14] Reantragoon R Corbett AJ Sakala IG Gherardin NA Furness JB Chen Z. Antigen-loaded MR1 tetramers define T cell receptor heterogeneity in mucosal-associated invariant T cells. J Exp Med. (2013) 210:2305–20. 10.1084/jem.20130958 24101382 PMC3804952

[15] Wong EB Ndung'u T Kasprowicz VO. The role of mucosal-associated invariant T cells in infectious diseases. Immunology. (2017) 150:45–54. 10.1111/imm.12673 27633333 PMC5341498

[16] Chiba A Murayama G Miyake S. Mucosal-associated invariant T cells in autoimmune diseases. Front Immunol. (2018) 9:1333. 10.3389/fimmu.2018.01333 29942318 PMC6004381

[17] Reantragoon R Boonpattanaporn N Corbett AJ McCluskey J. Mucosal-associated invariant T cells in clinical diseases. Asian Pac J Allergy Immunol. (2016) 34:3–10. 26994620

[18] Croxford JL Miyake S Huang YY Shimamura M Yamamura T. Invariant V(alpha)19i T cells regulate autoimmune inflammation. Nat Immunol. (2006) 7:987–94. 10.1038/ni1370 16878136

[19] Ruijing X Mengjun W Xiaoling Z Shu P Mei W Yingcheng Z. Jalpha33+ MAIT cells play a protective role in TNBS induced intestinal inflammation. Hepatogastroenterology. (2012) 59:762–7. 10.5754/hge11432 22115767

[20] Rouxel O Da Silva J Beaudoin L Nel I Tard C Cagninacci L. Cytotoxic and regulatory roles of mucosal-associated invariant T cells in type 1 diabetes. Nat Immunol. (2017) 18:1321–31. 10.1038/ni.3854 28991267 PMC6025738

[21] Kawano S Lin Q Amano H Kaneko T Nishikawa K Tsurui H. Phenotype conversion from rheumatoid arthritis to systemic lupus erythematosus by introduction of Yaa mutation into FcgammaRIIB-deficient C57BL/6 mice. Eur J Immunol. (2013) 43:770–8. 10.1002/eji.201243057 23280344

[22] Corbett AJ Eckle SB Birkinshaw RW Liu L Patel O Mahony J. T-cell activation by transitory neo-antigens derived from distinct microbial pathways. Nature. (2014) 509:361–5. 10.1038/nature13160 24695216

[23] Serriari NE Eoche M Lamotte L Lion J Fumery M Marcelo P. Innate mucosal-associated invariant T (MAIT) cells are activated in inflammatory bowel diseases. Clin Exp Immunol. (2014) 176:266–74. 10.1111/cei.12277 24450998 PMC3992039

[24] Cho YN Kee SJ Kim TJ Jin HM Kim MJ Jung HJ. Mucosal-associated invariant T cell deficiency in systemic lupus erythematosus. J Immunol. (2014) 193:3891–901. 10.4049/jimmunol.1302701 25225673

[25] Gracey E Qaiyum Z Almaghlouth I Lawson D Karki S Avvaru N. IL-7 primes IL-17 in mucosal-associated invariant T (MAIT) cells, which contribute to the Th17-axis in ankylosing spondylitis. Ann Rheum Dis. (2016) 75:2124–32. 10.1136/annrheumdis-2015-208902 27165176

[26] Haga K Chiba A Shibuya T Osada T Ishikawa D Kodani T. MAIT cells are activated and accumulated in the inflamed mucosa of ulcerative colitis. J Gastroenterol Hepatol. (2016) 31:965–72. 10.1111/jgh.13242 26590105

[27] Cassese G Arce S Hauser AE Lehnert K Moewes B Mostarac M. Plasma cell survival is mediated by synergistic effects of cytokines and adhesion-dependent signals. J Immunol. (2003) 171:1684–90. 10.4049/jimmunol.171.4.1684 12902466

[28] Radbruch A Muehlinghaus G Luger EO Inamine A Smith KG Dorner T. Competence and competition: the challenge of becoming a long-lived plasma cell. Nat Rev Immunol. (2006) 6:741–50. 10.1038/nri1886 16977339

[29] Crispin JC Keenan BT Finnell MD Bermas BL Schur P Massarotti E. Expression of CD44 variant isoforms CD44v3 and CD44v6 is increased on T cells from patients with systemic lupus erythematosus and is correlated with disease activity. Arthritis Rheum. (2010) 62:1431–7. 10.1002/art.27385 20213807 PMC2879041

[30] Crispin JC Oukka M Bayliss G Cohen RA Van Beek CA Stillman IE. Expanded double negative T cells in patients with systemic lupus erythematosus produce IL-17 and infiltrate the kidneys. J Immunol. (2008) 181:8761–6. 10.4049/jimmunol.181.12.8761 19050297 PMC2596652

[31] Fossati L Takahashi S Merino R Iwamoto M Aubry JP Nose M. An MRL/MpJ-lpr/lpr substrain with a limited expansion of lpr double-negative T cells and a reduced autoimmune syndrome. Int Immunol. (1993) 5:525–32. 10.1093/intimm/5.5.525 8318455

[32] Kato H Perl A. Mechanistic target of rapamycin complex 1 expands Th17 and IL-4+ CD4-CD8- double-negative T cells and contracts regulatory T cells in systemic lupus erythematosus. J Immunol. (2014) 192:4134–44. 10.4049/jimmunol.1301859 24683191 PMC3995867

[33] Mizui M Koga T Lieberman LA Beltran J Yoshida N Johnson MC. IL-2 protects lupus-prone mice from multiple end-organ damage by limiting CD4-CD8- IL-17-producing T cells. J Immunol. (2014) 193:2168–77. 10.4049/jimmunol.1400977 25063876 PMC4135016

[34] Patel O Kjer-Nielsen L Le Nours J Eckle SB Birkinshaw R Beddoe T. Recognition of vitamin B metabolites by mucosal-associated invariant T cells. Nat Commun. (2013) 4:2142. 10.1038/ncomms3142 23846752

[35] Eckle SB Birkinshaw RW Kostenko L Corbett AJ McWilliam HE Reantragoon R. A molecular basis underpinning the T cell receptor heterogeneity of mucosal-associated invariant T cells. J Exp Med. (2014) 211:1585–600. 10.1084/jem.20140484 25049336 PMC4113946

[36] Crow MK Type I interferon in the pathogenesis of lupus. J Immunol. (2014) 192:5459–68. 10.4049/jimmunol.1002795 24907379 PMC4083591

[37] Novak J Dobrovolny J Novakova L Kozak T. The decrease in number and change in phenotype of mucosal-associated invariant T cells in the elderly and differences in men and women of reproductive age. Scand J Immunol. (2014) 80:271–5. 10.1111/sji.12193 24846411

[38] Bennett MS Trivedi S Iyer AS Hale JS Leung DT Human mucosal-associated invariant T (MAIT) cells possess capacity for B cell help. J Leukoc Biol. (2017) 102:1261–9. 10.1189/jlb.4A0317-116R 28807929 PMC5636046

[39] Wen L Roberts SJ Viney JL Wong FS Mallick C Findly RC. Immunoglobulin synthesis and generalized autoimmunity in mice congenitally deficient in alpha beta(+) T cells. Nature. (1994) 369:654–8. 10.1038/369654a0 8208291

[40] Huang Y Heiser RA Detanico TO Getahun A Kirchenbaum GA Casper TL. gammadelta T cells affect IL-4 production and B-cell tolerance. Proc Natl Acad Sci USA. (2015) 112:E39–48. 10.1073/pnas.1415107111 25535377 PMC4291655

[41] Rezende RM Lanser AJ Rubino S Kuhn C Skillin N Moreira TG. gammadelta T cells control humoral immune response by inducing T follicular helper cell differentiation. Nat Commun. (2018) 9:3151. 10.1038/s41467-018-05487-9 30089795 PMC6082880

[42] Vomhof-DeKrey EE Yates J Leadbetter EA. Invariant NKT cells provide innate and adaptive help for B cells. Curr Opin Immunol. (2014) 28:12–7. 10.1016/j.coi.2014.01.007 24514004 PMC4346131

[43] Shen L Zhang H Caimol M Benike CJ Chakravarty EF Strober S. Invariant natural killer T cells in lupus patients promote IgG and IgG autoantibody production. Eur J Immunol. (2015) 45:612–23. 10.1002/eji.201444760 25352488 PMC4324163

[44] Munroe ME Lu R Zhao YD Fife DA Robertson JM Guthridge JM. Altered type II interferon precedes autoantibody accrual and elevated type I interferon activity prior to systemic lupus erythematosus classification. Ann Rheum Dis. (2016) 75:2014–21. 10.1136/annrheumdis-2015-208140 27088255 PMC4959992

[45] Hayashi E Chiba A Tada K Haga K Kitagaichi M Nakajima S. Involvement of mucosal-associated Invariant T cells in ankylosing spondylitis. J Rheumatol. (2016) 43:1695–703. 10.3899/jrheum.151133 27370879

[46] Hevia A Milani C Lopez P Cuervo A Arboleya S Duranti S. Intestinal dysbiosis associated with systemic lupus erythematosus. MBio. (2014) 5:e01548–14. 10.1128/mBio.01548-14 25271284 PMC4196225

[47] Zhang H Liao X Sparks JB Luo XM. Dynamics of gut microbiota in autoimmune lupus. Appl Environ Microbiol. (2014) 80:7551–60. 10.1128/AEM.02676-14 25261516 PMC4249226

