# Supplementary material for: A Critical Role for Mucosal-Associated Invariant T Cells as Regulators and Therapeutic Targets in Systemic Lupus Erythematosus
Source: Front Immunol. 2019 Nov 29;10:2681. doi: 10.3389/fimmu.2019.02681 (PMC6895065; doi:10.3389/fimmu.2019.02681)
Supplement: Supplementary file 1 [file Table_1.pdf]

Supplementary Table 1

**Nephritis class**	**II**	**III**	**IV**	**V**	**II+V**	**III+V**
n	1	2	6	6	1	3
Tubulointerstitium	0/1	2/2	4/6	0/6	0/1	0/3
Glomeruli	0/1	1/2	5/6	0/6	0/1	0/3

Supplementary table 1. Infiltration of $CD3^+$ cells into the glomeruli and tubulointerstitium of lupus nephritis patients. Nineteen kidney samples were analyzed using confocal microscopy. Data represent the number of samples containing $CD3^+$ cells/the number of analyzed samples in each class. The infiltration of $CD3^+$ cells was observed in kidney samples with class III and class IV nephritis.
