# Supplementary material for: A Critical Role for Mucosal-Associated Invariant T Cells as Regulators and Therapeutic Targets in Systemic Lupus Erythematosus
Source: Front Immunol. 2019 Nov 29;10:2681. doi: 10.3389/fimmu.2019.02681 (PMC6895065; doi:10.3389/fimmu.2019.02681)
Supplement: Supplementary file 2 [file Presentation_1.pdf]

Supplementary Figure 1

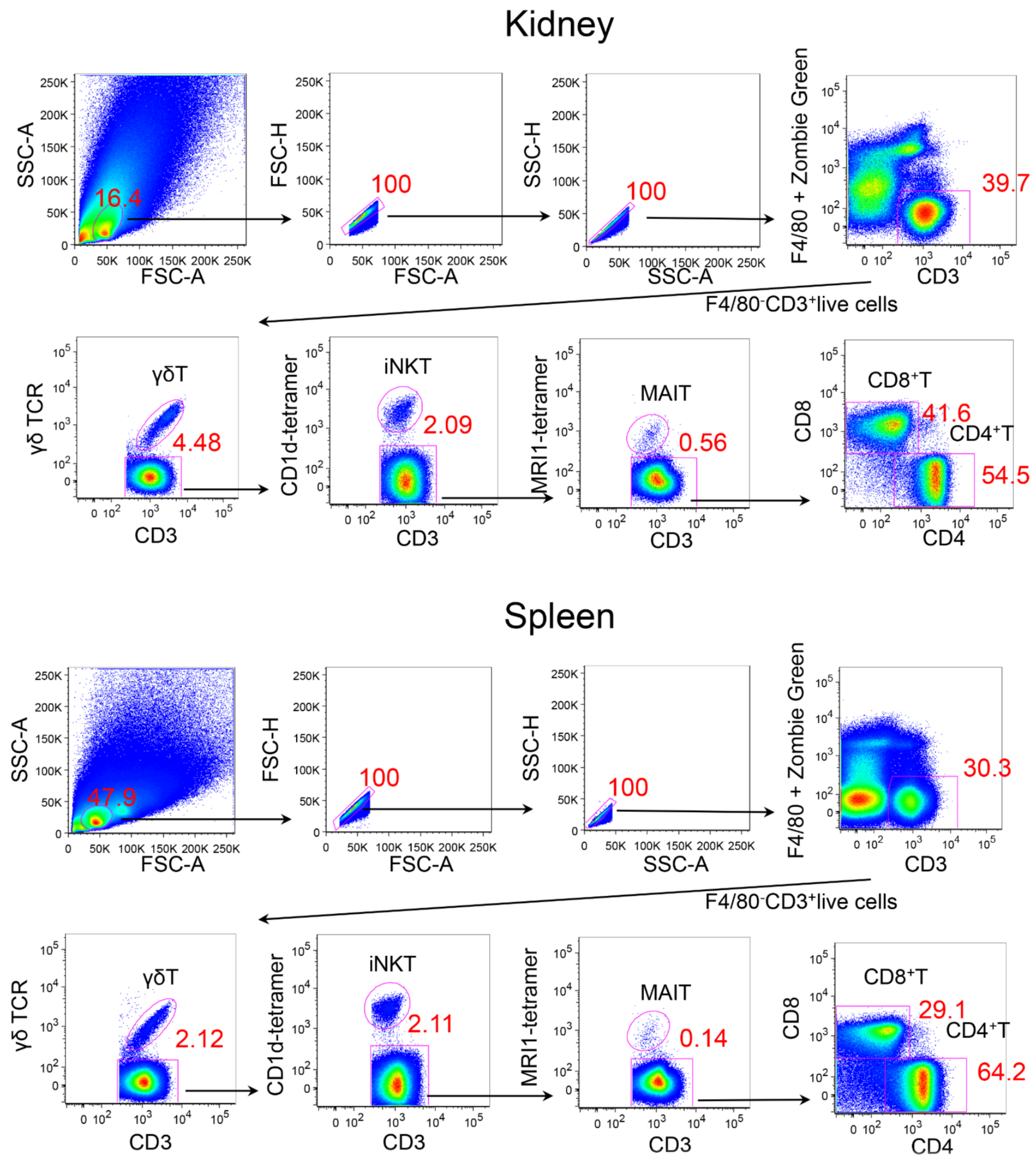

Supplementary Figure 1. Gating strategy for the flow cytometric analysis of innate T cells and T cells in the spleen and kidneys of FcγRIIb$^{-/-}$*Yaa* mice.

Lymphocytes were gated using a forward scatter area (FSC-A) versus side scatter area (SSC-A). Single cells (singlets) were selected by using FSC-A versus FSC-height (FSC-H) and SSC-A versus SSC-height (SSC-H) plots. Zombie green positive dead cells and F4/80 positive cells were excluded to gate out cells that bind to antibodies and tetramers non-specifically. Among CD3 positive cells, γδT cells (TCRγδ^{+}), iNKT cells (TCRγδ^{-}CD1d/PBS-57 tetramer^{+}), and MAIT cells (TCRγδ^{-} CD1d/PBS-57 tetramer^{-}MR1/5-OP-RU tetramer^{+}) were gated as shown.
