# Supplementary material for: A Critical Role for Mucosal-Associated Invariant T Cells as Regulators and Therapeutic Targets in Systemic Lupus Erythematosus
Source: Front Immunol. 2019 Nov 29;10:2681. doi: 10.3389/fimmu.2019.02681 (PMC6895065; doi:10.3389/fimmu.2019.02681)
Supplement: Supplementary file 3 [file Presentation_2.PDF]

frontiers

# *Supplementary Figure 2*

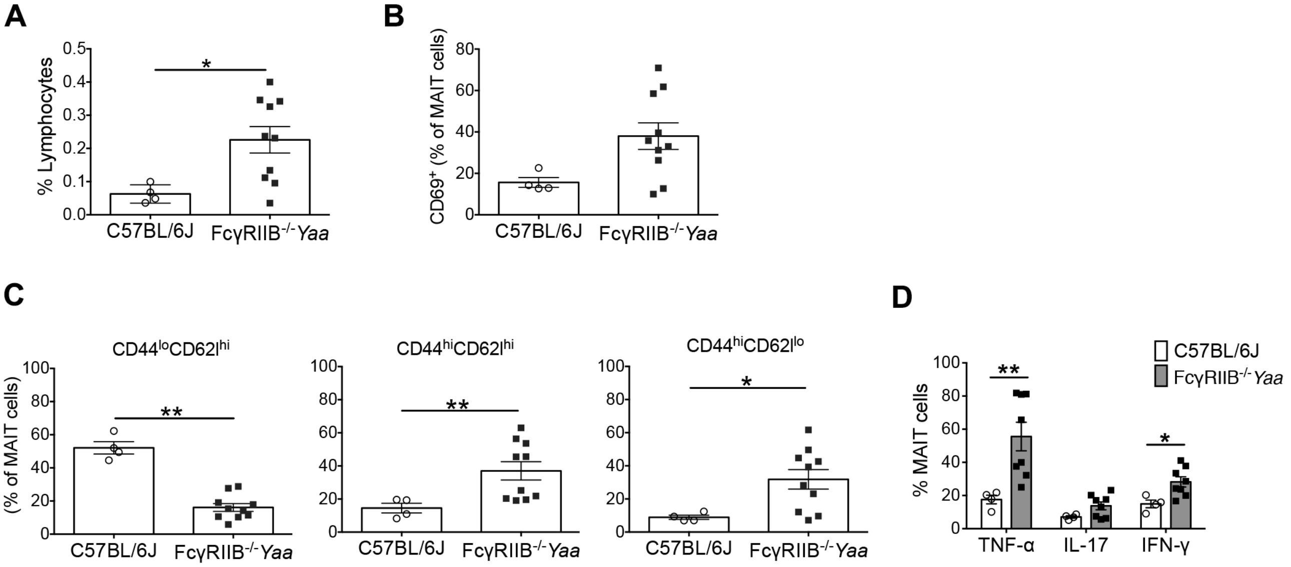


**Supplementary Figure 2. Flow cytometric analysis of MAIT cells from FcγRIIb$^{-/-}$*Yaa* and C57BL/6J mice.** (A-D) Frequency and phenotype of MAIT cells in spleens from C57BL/6J and FcγRIIb$^{-/-}$*Yaa* mice at 2 months of age. Data from MR1$^{+/+}$ FcγRIIb$^{-/-}$*Yaa* mice in experiments in Figure 3 were compared to those from C57BL/6J mice. (A) Flow cytometric evaluation of the frequencies of MAIT cells (A) and CD69$^{+}$, naïve, central memory and effector memory cells among MAIT cells (B, C). (D) Flow cytometric evaluation of the frequencies of the indicated cytokine-producing cells among MAIT cells upon stimulation with PMA and ionomycin. Values in (A-D) are shown as the mean ± SEM. Each symbol represents data from an individual mouse. *p* values in (A-D) were determined by two-tailed Mann-Whitney *U*-test (**$p < 0.01$, *$p < 0.05$).
