# Supplementary material for: A Critical Role for Mucosal-Associated Invariant T Cells as Regulators and Therapeutic Targets in Systemic Lupus Erythematosus
Source: Front Immunol. 2019 Nov 29;10:2681. doi: 10.3389/fimmu.2019.02681 (PMC6895065; doi:10.3389/fimmu.2019.02681)
Supplement: Supplementary file 5 [file Presentation_4.PDF]

Supplementary Figure 4

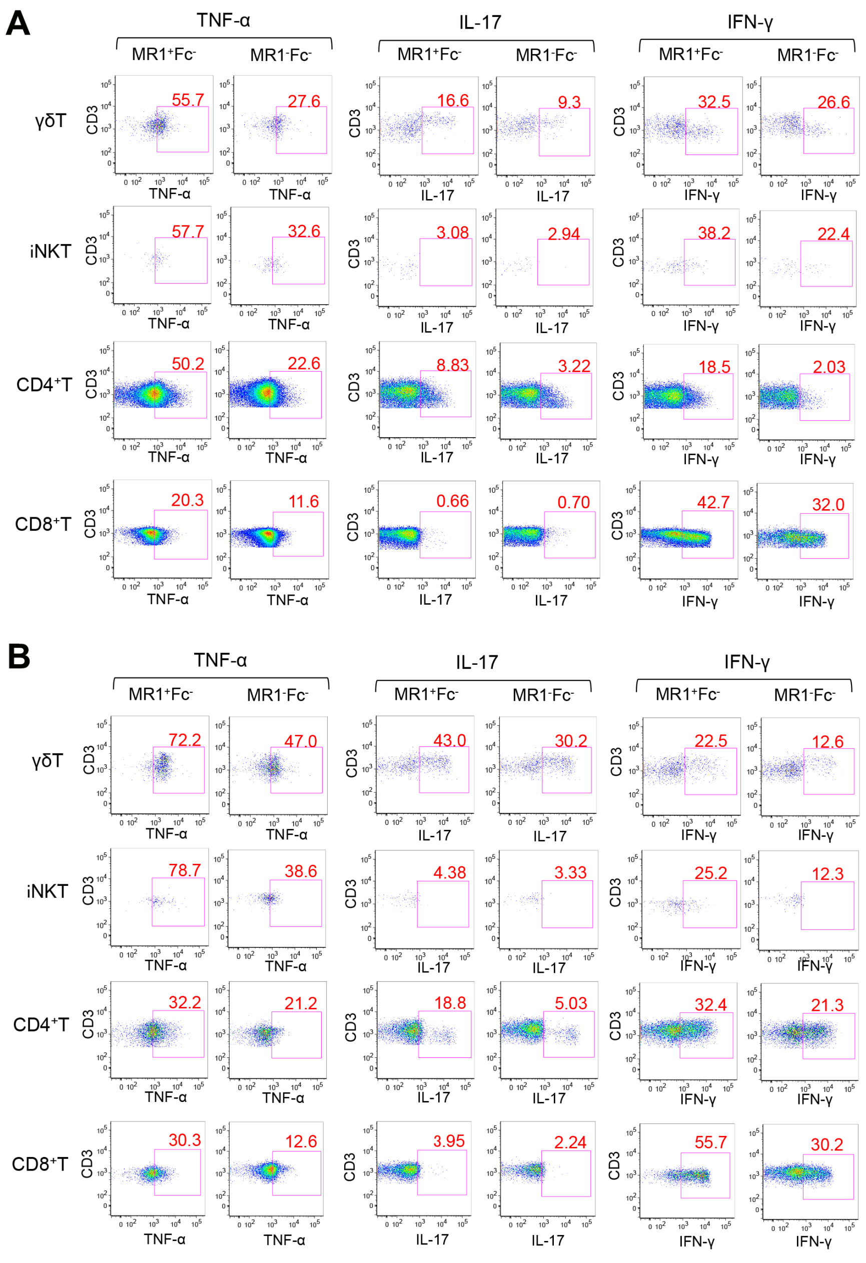

Supplementary Figure 4. Representative flow cytometry profiles of cytokine staining in innate T cells and T cells from MR1$^{+/+}$(MR1^{+}Fc^{-}) and MR1$^{-/-}$ FcγRIIb$^{-/-}$*Yaa* (MR1^{-}Fc^{-}) mice. Representative flow cytometry profiles of the indicated cytokine-producing cells among γδT, iNKT, CD4^{+}T, and CD8^{+}T cells upon stimulation with phorbol 12-myristate 13-acetate (PMA) and ionomycin in the spleen (A) and kidneys (B) of MR1$^{+/+}$ (MR1^{+}Fc^{-}) (n=15) and MR1$^{-/-}$ FcγRIIb$^{-/-}$*Yaa* (MR1^{-}Fc^{-}) mice. Each innate T cell and T cell subset was gated as shown in the gating strategy in Supplementary Figure 1.
