# Supplementary material for: A Critical Role for Mucosal-Associated Invariant T Cells as Regulators and Therapeutic Targets in Systemic Lupus Erythematosus
Source: Front Immunol. 2019 Nov 29;10:2681. doi: 10.3389/fimmu.2019.02681 (PMC6895065; doi:10.3389/fimmu.2019.02681)
Supplement: Supplementary file 6 [file Presentation_5.PDF]

Supplementary Figure 5

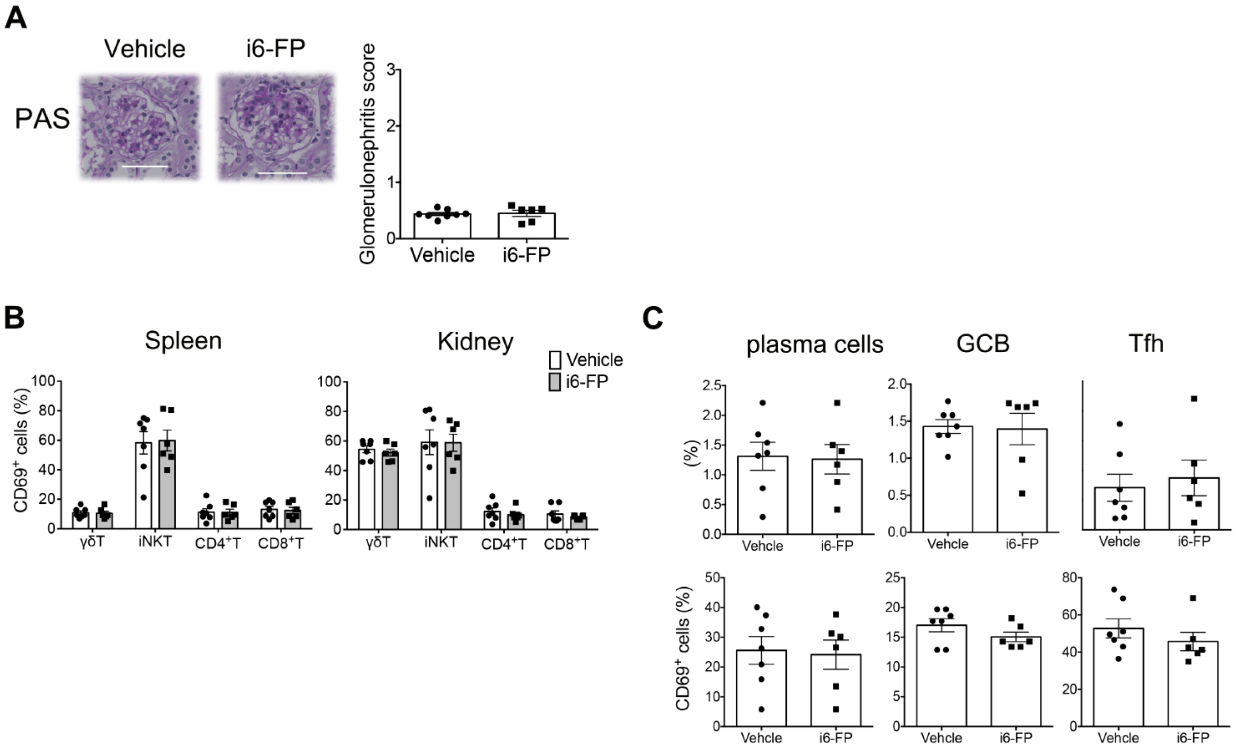

Supplementary Figure 5. i6-FP administration failed to suppress lupus in MR1$^{-/-}$ FcγRIIb$^{-/-}$*Yaa* mice. MR1$^{-/-}$FcγRIIb$^{-/-}$*Yaa* (MR1^{-}Fc^{-}) mice were treated orally with i6-FP (n=6) or control vehicle (n=7) three times weekly starting at four weeks of age. (A) Histopathological findings of glomeruli. Representative images of kidney sections and histopathological scores are shown. Scale bars, 50 µm. Representative images of frozen kidney sections stained with anti-mouse IgG and C3 in glomeruli (scale bar, 50 µm). (B) CD69 expression of innate T cells and T cells in the spleen and kidneys from MR1^{-}Fc^{-} mice at 2 months of age were analyzed by flow cytometry. Percentages of CD69^{+} cells among γδT cells, iNKT cells, MAIT cells, CD4^{+}T cells, and CD8^{+}T cells are shown. (C) Flow cytometric evaluation of the frequencies of plasma cells, germinal center B cells (GCB), and T follicular helper cells (Tfh) and percentages of CD69 positivity among these B and T cell subsets.
