# Supplementary material for: A Critical Role for Mucosal-Associated Invariant T Cells as Regulators and Therapeutic Targets in Systemic Lupus Erythematosus
Source: Front Immunol. 2019 Nov 29;10:2681. doi: 10.3389/fimmu.2019.02681 (PMC6895065; doi:10.3389/fimmu.2019.02681)
Supplement: Supplementary file 7 [file Presentation_6.PDF]

Supplementary Figure 6

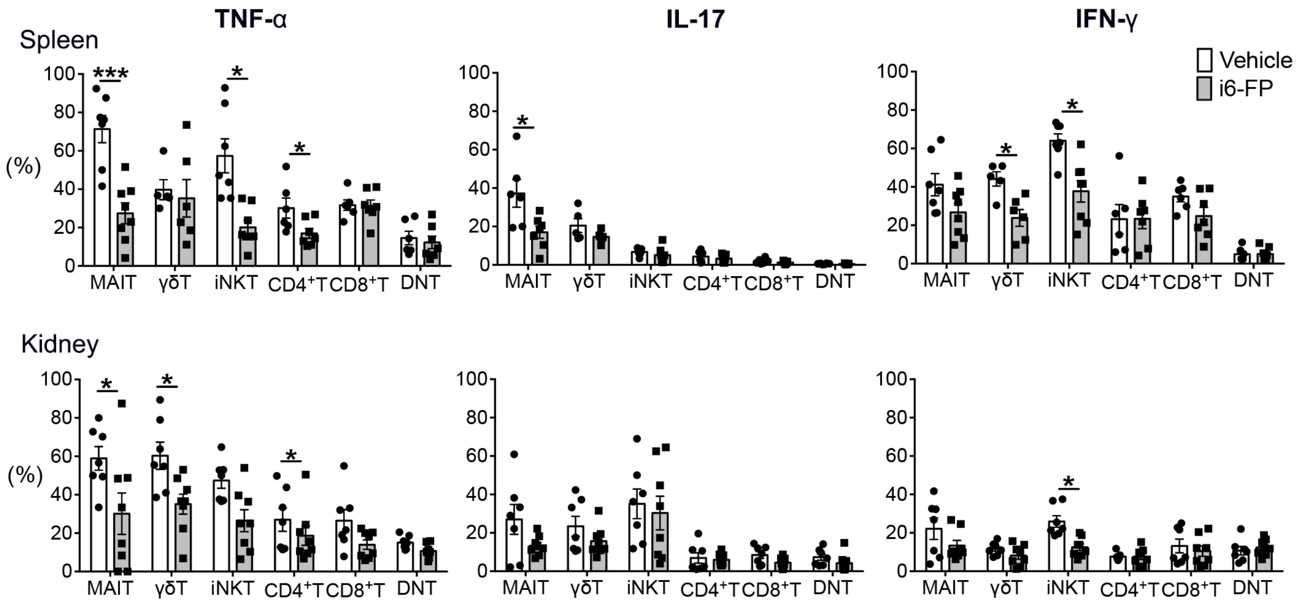

Supplementary Figure 6. i6-FP administration reduces innate T and T cell responses lupus in FcγRIIb$^{-/-}$*Yaa* mice. Flow cytometric evaluation of the frequencies of the indicated cytokine-producing cells among MAIT, γδT, iNKT, CD4^{+}T, CD8^{+}T and CD4^{-}CD8^{-} double-negative (DN) T cells upon stimulation with PMA and ionomycin. Each symbol represents data from individual mice. *p*-values were determined by two-tailed Mann-Whitney *U*-test (*****p* < 0.0001, ****p* < 0.001, ***p* < 0.01, **p* < 0.05).
