# Supplementary material for: A Critical Role for Mucosal-Associated Invariant T Cells as Regulators and Therapeutic Targets in Systemic Lupus Erythematosus
Source: Front Immunol. 2019 Nov 29;10:2681. doi: 10.3389/fimmu.2019.02681 (PMC6895065; doi:10.3389/fimmu.2019.02681)
Supplement: Supplementary file 8 [file Presentation_7.pdf]

Supplementary Figure 7

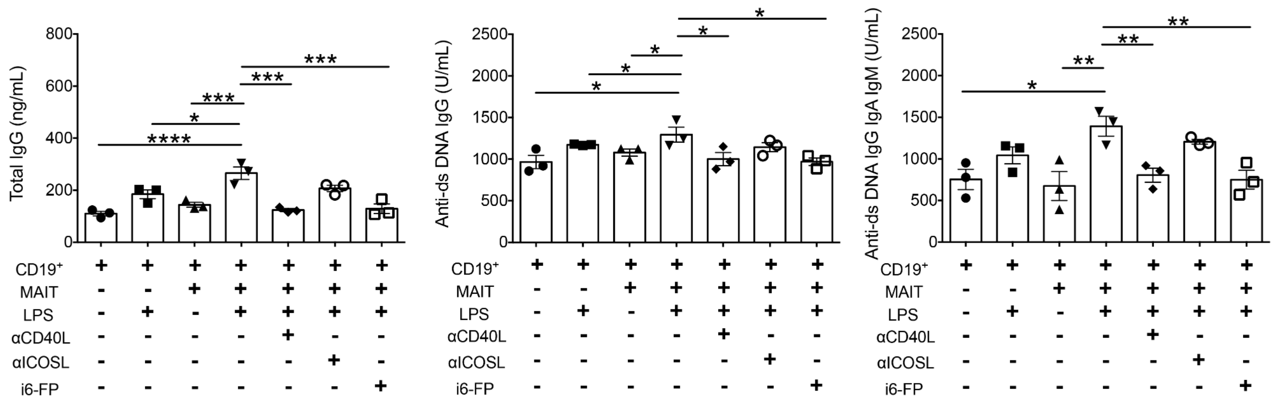

Supplementary Figure 7. MAIT cells enhance autoantibody production by B cells when cultured at a ratio of 1:1.

B cells from FcγRIIb$^{-/-}$*Yaa* mice were stimulated with lipopolysaccharide (LPS) in the presence or absence of MAIT cells at a ratio of 1:1, blocking antibodies against CD40L, ICOS or i6-FP (10 µM). ELISA results of total IgG, and anti-dsDNA IgG and anti-ds DNA IgG+A+M in the culture supernatants. Each symbol represents data from each experiment. *p*-values were determined by two-tailed, one-way ANOVA followed by Tukey's multiple comparison tests (****$p < 0.0001$, ***$p < 0.001$, **$p < 0.01$, *$p < 0.05$).
