# Supplementary material for: A Critical Role for Mucosal-Associated Invariant T Cells as Regulators and Therapeutic Targets in Systemic Lupus Erythematosus
Source: Front Immunol. 2019 Nov 29;10:2681. doi: 10.3389/fimmu.2019.02681 (PMC6895065; doi:10.3389/fimmu.2019.02681)
Supplement: Supplementary file 4 [file Presentation_3.pdf]

frontiers

Supplementary Figure 3

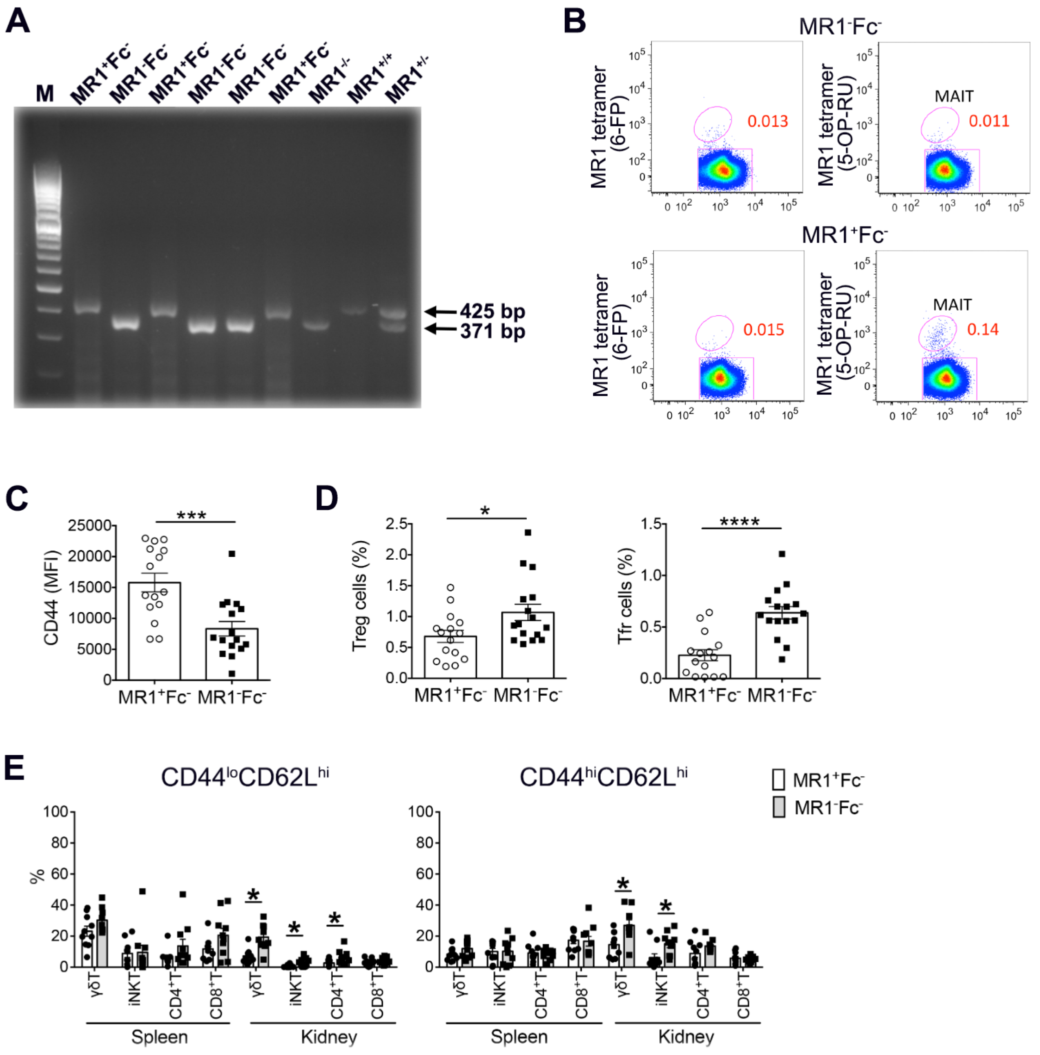

Supplementary Figure 3. Genotyping of MR1 knockout and flow cytometric analysis of B and T cell subsets in MR1$^{+/+}$ (MR1^{+}Fc^{-}) and MR1$^{-/-}$ FcγRIIb$^{-/-}$*Yaa* (MR1^{-}Fc^{-}) mice. (A) PCR Genotyping of MR1$^{-/-}$ FcγRIIb$^{-/-}$*Yaa* (MR1^{-}Fc^{-}) and MR1$^{+/+}$ FcγRIIb$^{-/-}$*Yaa* (MR1^{+}Fc^{-}) mice and MR1$^{-/-}$, MR1$^{+/+}$ and MR1$^{+/-}$ C57BL/6J mice using DNA isolated from tail biopsies. A representative agarose gel electrophoresis image is shown. M, DNA markers. (B) Representative flow cytometry images of F4/80^{-}CD3^{+}γδTCR^{-}CD1d/PBS-57 tetramer^{-} lymphocytes in the spleen from MR1^{+} and MR1^{-}Fc^{-} mice. (C) Mean fluorescence intensity (MFI) of CD44 on plasma cells. (D) Frequencies of T regulatory cells (Treg) (CD3^{+}CD4^{+}Foxp3^{+}CD25^{+}) and T follicular regulatory cells (Tfr) (CD3^{+}CD4^{+}CD69^{+}Foxp3^{+}CD25^{lo}CXCR5^{+}Bcl6^{+}). (E) Frequencies of naïve T cells (CD44^{lo}CD62L^{hi}) and central memory T cells (CD44^{hi}CD62L^{hi}) among γδT cells, iNKT cells, CD4^{+}T cells and CD8^{+}T cells. Each symbol represents the value of one individual. Values in C-E are shown as the mean ± SEM. *p*-values were determined by two-tailed Mann-Whitney *U*-test (*$p < 0.05$).
